# Utilisation of digital educational technology amongst undergraduate nursing students in the Western Cape

**DOI:** 10.4102/hsag.v31i0.3321

**Published:** 2026-05-30

**Authors:** Million Bimerew, Victoire Ticha, Jenifer Chipps

**Affiliations:** 1 School of Nursing, Faculty of Community and Health Sciences, University of the Western Cape, Cape Town, South Africa

**Keywords:** digital educational technology, nursing student, utilisation, undergraduate, Western Cape

## Abstract

**Background:**

Despite increased integration of digital technologies in higher education, nursing students face major barriers in resource-limited settings.

**Aim:**

The study investigated the utilisation of digital educational technologies amongst undergraduate nursing students in the Western Cape.

**Setting:**

The study was conducted at the school of nursing at a higher education institution in the Western Cape.

**Methods:**

A descriptive cross-sectional survey design with a self-administered questionnaire was used to collect data from a sample of 253 participants. Descriptive analysis was conducted to determine the frequencies and percentages; the mean score and Chi-square test were conducted to determine the association between the variables.

**Results:**

Nearly 90% of participants had experience in the use of digital educational technology, 94.5%, learning management system and Facebook, and 93.7% used PowerPoint, communication and collaboration tools. The gender differences in technology use are consistent with the literature. The results suggest female respondents’ frequent use of social networks. Male respondents reported more frequent use of bibliographic and web conferencing technologies. Although overall use was very low, gender remains an important factor in understanding technology use. In contrast, blogs, discussion forums and immersive technologies were amongst the least utilised educational technologies.

**Conclusion:**

Most nursing students use basic digital tools like learning management systems, Facebook and PowerPoint, but advanced tools such as blogs, forums and immersive technologies are underused, revealing a gap in digital competence that limits collaborative learning and clinical simulation.

**Contribution:**

This study provides evidence on digital technology use amongst undergraduate nursing students, showing frequent use of basic tools but limited engagement with advanced, interactive technologies.

## Introduction

Nursing education is evolving due to technological advancements, and training institutions are expected to incorporate best practices and make sure that students are interested and motivated with the educational use of technology. In order to interact with the technology-mediated learning environment, nursing students must acquire digital educational technology competencies, which cannot be presumed to have been a part of their prior education. Technology is said to improve students’ motivation and focus whilst supporting student-centred learning strategies (Owusu-Agyeman & Larbi-Siaw 2018). It enables instructional delivery beyond the confines of the traditional classroom, thereby overcoming temporal and geographical limitations. In addition, it fosters self-directed and inquiry-driven learning, promotes collaborative engagement and cultivates lifelong learning habits (Forde et al. 2024). Furthermore, it enhances critical thinking skills and supports the effective integration of theoretical knowledge into clinical practice. These competencies are fundamental to the nursing profession. Leonardsen et al. (2023) reported that students possessing advanced digital competencies demonstrated superior academic performance, attributable to their ability to efficiently retrieve information and complete online coursework. In a similar vein, Ibrahim and Aldawsari (2023) identified a significant positive association between digital competence and academic achievement in hybrid learning settings. Collins (2024) reported that nursing students demonstrating higher levels of digital competence showed greater engagement with online learning platforms, including active participation in virtual discussions and collaborative group projects. This heightened involvement was associated with increased academic motivation, as students developed greater confidence in their ability to use digital tools effectively. Moreover, access to a variety of digital learning resources, such as instructional videos, interactive quizzes and simulation activities, offered flexible and engaging learning experiences that further strengthened student motivation. Similarly, Haleem et al. (2022) found that students with well-developed digital skills were more inclined to engage in self-directed learning activities, including reviewing online tutorials and taking part in virtual simulations. Such practices were linked to enhanced intrinsic motivation, as students assumed greater responsibility and autonomy in managing their own educational experiences.

Furthermore, it allows for more personalised and flexible learning experiences, it plays a critical role in skills development, increases engagement in practical real-world scenarios and in a safe, controlled environment (Graham, Allen & Ure 2015; Tomas, Thomas & Carey 2020). However, the development of such competencies remains challenging for many nursing students, often due to insufficient access to technological resources or inadequate training opportunities.

Despite these promising potential benefits, nursing institutions in low-income countries face challenges in effectively incorporating digital technologies due to infrastructural constraints, the need for educator training and limited resources for technology implementation (Spector 2016). Garzón-Artacho et al. (2021) emphasised that limited digital proficiency constitutes a substantial barrier, particularly in online courses that demand active engagement with digital tools for learning, communication and collaborative activities. Many nursing schools lack the necessary hardware and software and reliable internet connectivity to support the integration of technology into their educational programmes (Leow, Lee & Cheong 2020). This digital divide limits access to the tools needed to implement a technology-enhanced curriculum. This lack of resources further exacerbates the disparity in educational quality between institutions in developed and developing countries (Graham et al. 2015).

Apart from the resource constraints in the use of digital educational technologies in nursing education, it provides challenges for student engagement and motivation. Nursing students often must balance demanding clinical placements and academic responsibilities, making it difficult for them to remain fully engaged with digital learning platforms (El-Sayed 2023). Whilst digital tools can offer more flexible and individualised learning experiences, they also pose the risk of student disengagement. Research has shown that students may have difficulty staying motivated and actively participating in online courses, especially in the absence of face-to-face interaction and hands-on experiences. In addition, not all students are comfortable with or prepared for digital learning. Some students may lack the necessary digital literacy skills to effectively navigate online platforms or may struggle with technology-related challenges such as poor internet access or inadequate devices (Spector 2016). Ineffective integration of educational technology, inadequate educators’ training in technology use and insufficient financial investment in technological development and maintenance have been consistently associated with poor learning outcomes in nursing education (Makhene, Nene & Mhlongo 2025). These can lead to a lack of confidence in using digital tools and, consequently, reduced learning outcomes. On the other hand, in many nursing educational settings, there is a deep-rooted belief that face-to-face learning is more effective than online or blended learning. This belief can create resistance to the adoption of digital tools, especially amongst senior educators who have been accustomed to traditional teaching methods for decades (Beyea & Nicoll 2020). Additionally, institutional resistance may arise due to the perceived complexity and cost of integrating new technologies into existing curricula. Nursing institutions may be reluctant to adopt new technologies unless they are assured of their effectiveness in improving learning outcomes and their alignment with institutional goals (Leow et al. 2020). Some nursing training institutions are wary of over-relying on technology, fearing that it might detract from essential hands-on, clinical learning experiences that are fundamental in nursing education. As nursing education is highly practice-oriented, some staff members may argue that virtual simulations cannot fully replicate the experience and learning that takes place in clinical settings (Beyea & Nicoll 2020). This scepticism about the efficacy of digital tools can contribute to slower adoption rates and limited use of these technologies in nursing education programmes. Thus, this study investigated the utilisation of digital educational technologies amongst undergraduate nursing students in the Western Cape.

## Research methods and design

### Study design

A descriptive cross-sectional survey design was employed to investigate the extent and patterns of digital educational technology utilisation amongst undergraduate nursing students. Data were collected using a structured, self-administered questionnaire, specifically developed to capture participants’ access to, frequency of use and perceived competence with various digital learning tools and platforms. The survey approach was selected to enable the systematic collection of standardised data from a defined student population, thereby facilitating quantitative analysis of trends, behaviours and associated factors related to the integration of digital educational technologies within undergraduate nursing education.

### Research setting

The study was conducted in one of the nursing schools at a higher education institution in the Western Cape that offers both undergraduate and postgraduate nursing programmes. This setting currently uses a blended learning approach to incorporate digital educational technologies in the learning and teaching modalities.

### Population and sample

The population for this study included 650 undergraduate nursing students enrolled in nursing programmes for 2024 at a selected higher education institution. Non-probability sampling was used to recruit participants from various academic years to ensure a broad perspective on digital educational technology utilisation. The sample size was calculated using Yamane’s sample size formula, *n = N/1 + N(e*^2^). The total calculated sample size was 286, using *p* = 50%, error = 5% and confidence level 95%, distributed across four year levels. All undergraduates from first year to fourth year nursing students registered in the 2024 academic year and having experience using digital educational technology as part of their coursework or clinical training were included. The exclusion criteria were all postgraduate students or those suspended for any academic or personal reasons.

### Data collection

The study will adopt an existing questionnaire developed from previously published literature (Kokol et al. 2006; Lloyd, Byrne & Mccoy 2012; Manning & Johnson 2011; Silveira & Cogo 2017). The survey tool was developed based on a review of existing literature on digital education utilisation in nursing education (Beyea & Nicoll 2020; Leow et al. 2020). The instrument was pre-tested on a small group of nursing students to assess clarity, reliability and validity. Cronbach’s alpha was calculated to determine the internal consistency of the survey items (Polit & Beck 2017). The Cronbach’s alpha was α = 0.854 for 58 items. The survey instrument included closed-ended and Likert-scale questions designed to assess the frequency, types and purposes of digital tools used by nursing students and perceived effectiveness and barriers to utilisation. The tool had previously been used in a similar survey study. The tools include a 5-point Likert-scale with 58 items within five dimensions (organisational tools, communications tools, presentation tools, learning assessment tools and identity transformation tools). Data were collected by the researchers using a structured self-administered questionnaire. The questionnaires were distributed to participating students during a scheduled 20-min academic break to minimise disruption to instructional activities. Completion of the survey instrument required approximately 10 min – 15 min. Data were collected in October 2024. Prior to participation, students were provided with detailed information regarding the purpose, procedures and voluntary nature of the study, and written informed consent was obtained from all respondents before they completed the questionnaire.

With respect to data management and confidentiality, all collected data were handled in accordance with established ethical and institutional guidelines. Electronic data were securely stored on a password-protected computer accessible only to the principal researcher. Hard copy questionnaires were retained in a locked filing cabinet to ensure restricted access. These measures were implemented to safeguard participant confidentiality and to maintain the integrity and security of the research data.

### Data analysis

Statistical package for social sciences developed by IBM (International Business Machines Corporation, Armonk, NY) was used to analyse the data. A descriptive statistic, including frequencies, percentages and mean scores to provide an overview of the patterns and trends related to Digital Educational Technology (DET) utilisation on individual item factors of digital technology device, frequency use of eLearning technology network, perceived benefits, constraints and implications to use of digital educational technologies. Nonparametric tests, such as Chi-square tests, were performed to examine potential differences in DET utilisation based on demographic variables such as academic year, gender and digital educational technology utilisation. The *p*-value < 0.05 was considered statistically significant.

### Ethical considerations

The study received ethical approval from the Humanities and Social Sciences Research Ethics Committee (HSSREC) of the University (Ref.HS24/4/14). This study adhered to ethical guidelines to ensure the protection of participants’ rights and confidentiality. Respondents were informed about the purpose of the study, and their participation was voluntary. Informed consent was obtained from each participant prior to data collection. The survey was anonymous to ensure that participants’ responses could not be traced back to them. Data were stored securely, and only the research team has access to the data. The study ethics was approved by the ethics committee of the institution to ensure compliance with ethical standards (Marshall & Rossman 2016).

## Results

### Characteristics of respondents

A total of 253 individuals participated in the survey of which 214 (84.6%) were female. Most respondents (172; 67.7%) were between the ages of 20 and 30. Respondents were distributed across the year levels, with the third-year students constituting the largest group with 85 respondents (33.5%), followed by the fourth-year students with 71 (28.0%), first-year students with 53 (20.9%) and second-year students with 45 (17.7%) as depicted in Table 1.

**TABLE 1:** Demographic characteristics of respondents (*N* = 253).

Characteristic	Frequency (*N* = 253)	
	*n*	%
**Gender**		
Female	214	84.6
Male	39	15.4
**Age (years)**		
< 20	74	29.1
20–30	172	67.7
> 31	7	2.8
**Year level**		
1st year	53	20.9
2nd year	45	17.7
3rd year	85	33.5
4th year	71	28.0
Experienced in the use of digital education technologies	223	89.8
Training course	35	13.8

### Utilisation patterns of digital educational technologies

Nearly all the respondents indicated that they have had experience in the use of digital educational technology (223, 89.8%), although only 35 (13.8%) indicated that they had done a course. The most frequently (frequent to very frequent) used digital educational technologies were the university’s official learning management system *Ikamva* (248, 94.5%, 3.8/4 ± 0.61), a learning organisation and management tool, followed by social networking platforms WhatsApp and Facebook (240, 94.5%, 3.74/4 ± 0.61), communication and collaboration tools, and PowerPoint (238, 93.7%, 3.69/4 ± 0.58), a learning content delivery tool (Table 2). Female respondents reported significantly more frequent use than male respondents for the use of social networks (3.78 ± 0.57 vs 3.52 ± 0.72, *U* = 2.9, *p* = 0.003), the use of PowerPoint (3.73 ± 0.56 vs 3.51 ± 0.68, *U* = 2.3, *p* = 0.023) and male respondents used bibliographic referencing (2.3 ± 1.2 vs 1.79 ± 1.22, *U* = 2.3, *p* = 0.025) and web conferencing more frequently (1.92 ± 1.3 vs 136 ± 1.18, *U* = 2.6, *p* = 0.010), although overall use was very low (1.45, 1.2). In contrast, blogs, discussion forums and immersive technologies were amongst the least utilised, with only 111 (43.7%) (0.91 ± 1.03), 87 (34.3%) and 57 (22.4%) (1.38 ± 1.14) reporting never using these technologies (Table 2). These findings identified that whilst core platforms like Ikamva and social media are widely adopted across the student population, more advanced or collaborative technologies, such as immersive tools and discussion forums, remain underutilised. Furthermore, usage patterns vary according to gender.

**TABLE 2:** The frequency of digital educational technology use.

Digital educational technology use tools	Mean	s.d.	Overall rank in use
**Communication and collaboration**			
Social network (WhatsApp, Facebook, etc.)	3.74	0.61	2
Instant messages and chats	3.42	0.97	4
Video calls	2.99	1.14	10
Web conference	1.44	1.22	16
Discussion forums	1.38	1.09	17
Bloggs	0.91	1.03	19
**Learning and content delivery**			
PowerPoint	3.69	0.58	3
Video	3.42	0.8	5
Audio	3.25	0.99	6
Tutorials	3.16	0.99	7
Virtual scenarios	1.78	1.21	15
Immersive technologies	1.18	1.13	18
**Learning organisation and management**			
Ikamva	3.8	0.49	1
Online schedules	2.44	1.09	12
Mind maps or graphic organisers	1.98	1.26	13
**Resource sharing and knowledge management**			
Image sharing	3.13	1.13	8
Research-based information	2.8	1.05	11
Bibliographic sharing	1.86	1.23	14
**Assessment and feedback**			
Online tests	3.01	0.95	9

s.d., standard deviation.

### Benefits of using digital educational technologies

Benefits were rated through agreement level with eight statements, with all statements receiving > 80 level of agreement (Table 3). The highest level of agreement was for the statement that digital educational technology facilitates the integration of diverse learning tools, such as PDF documents, web links and educational applications – into the learning process (252; 99.2%) followed by 243 respondents (95.7%) indicating that it enables access to educational content regardless of time or location, allowing learners to engage with materials at their own pace and convenience (Table 3). There were no significant differences in agreement with the statements except for that digital educational technology improves the presentation of nursing care, where male (38; 97.4%) respondents had a higher level of agreement than female respondents (180; 84.5%), (χ^2^ = 4.721, *p* = 0.030), although both had more than 80% of the respondents agreeing.

**TABLE 3:** Agreement with perceived benefits of using digital educational technologies.

Benefits statements	*n*	%
It allows the integration of multiple learning tools (pdf, links, app, amongst others)	252	99.2
It allows access to educational content without time or space limits; Low cost	243	95.7
Improves the quality of the teaching-learning process	238	93.7
Improves students’ preparation for applying theoretical content to practice	227	89.4
Improves the student’s ability to analyse, synthesise and think critically	226	89.0
Promote student accountability	220	68.6
Enhances multidisciplinary work	220	86.6
Improves the presentation of nursing care	219	86.2

### Constraints of using digital educational technologies

Perceptions about constraints using digital educational technology were rated through agreement level with 11 statements, with agreement levels ranging from 237 (93.3%) respondents agreeing with the constraint of technical issues, such as network failures, server overloads, power outages and unstable internet connectivity, to 79 (31.3%) agreeing with fear of technology (Table 4). In addition, three-quarters (198, 78%) agreed that reduced physical interaction with peers was a limiting factor. A statistically significant difference was observed between genders regarding perceptions of depersonalisation associated with the use of digital educational technology, with more male respondents (21, 53.8%) agreeing with this statement compared to female respondents (63, 29.7%) (χ^2^ = 8.61, *p* = 0.003). Similarly, more male respondents (26, 692%) agreed with this statement than female respondents (89, 42.2%) (χ^2^ = 8.61, *p* = 0.003). (χ^2^ = 9.69, *p* = 0.003).

**TABLE 4:** Agreement with perceived constraints in using digital educational technology.

Constraints statements	*n*	%
Technical difficulties (network failure, server overload, power outage, unstable internet connectivity)	237	93.3
Less physical contact with students	198	78.0
Constant updating and innovation of digital educational technology	180	70.9
Moving away from traditional teaching models	175	69.9
Difficulty in obtaining visual feedback	144	56.7
Lack of regulation in the use of digital educational technology	127	50.0
Lack of infrastructure and technologies	121	47.6
Resistance to the use of digital educational technology	117	46.1
Depersonalisation (negative attitudes) towards teaching	85	33.6
Difficulty in managing time and content	84	33.1
Fear of technology	79	31.1

### Implications of the use of digital educational technologies on teaching and learning

Agreement on the implications of the use of digital educational technologies for teaching and learning was measured through 18 statements: eight on simulation, five on learning and five on learning skills were rated (Table 5).

**TABLE 5:** Implications of using digital educational technology for simulation, stimulation and learning skills.

Implications	*n*	%
Implications of DET use for simulation		
Possibility of using software in simulation scenarios	231	90.9
Possibility to repeat simulations until competent	229	90.2
Promotion of meaningful learning	228	89.8
New technologies, such as clinical simulation technology, are embedded in the learning processes	228	89.8
Ensures better practical performance for the student	200	78.7
Laboratories are well utilised in using technology to impart education	186	73.2
Skills laboratory is well-equipped and up to date with the latest technology	172	67.7
Virtual or augmented reality for clinical skills are embedded in the learning processes	163	64.2
**Implications for stimulation of learning**		
The existence of multiple learning tools makes learning more stimulating	244	96.1
Stimulates students’ independence	236	92.9
Allows to personalise the learning and teaching	233	91.7
Arouses students’ curiosity	228	89.8
Improve use of theoretical content	227	89.4
**Implications for learning skills**		
Stimulate self-learning	244	96.1
Safe practice environment allows errors that lead to improved technical execution	233	91.7
Improve performance in the execution of the clinical skills procedure	233	91.7
Make learning less monotonous/repetitive	206	81.1
Decreases anxiety when performing a clinical simulation procedure	198	78.0

DET, digital educational technology.

#### Implications for simulation

All statements had > 60% agreement, with more than 80% of the respondents agreeing with four of the statements. The highest level of agreement was for having the possibility to repeat simulations (229, 90.2%), and for the possibility of using software in simulation scenarios (231, 90.9%), with 229 (90.2%) participants affirming this benefit in a separate item, reinforcing the perceived value of repetition in skills acquisition. Agreement levels on the embedding of these technologies in the skills laboratories were between 60% and 73% (Table 5). No statistically significant differences were observed.

#### Implications for learning

The findings strongly support the role of digital educational technology in stimulating both learning and the development of learning skills, with all agreement levels above 89% (Table 5). Respondents agreed that using digital educational technology made learning more stimulating (244, 96.1%), stimulated independence (236, 92.9%), allowed for personalisation (233, 91.7%), stimulated curiosity (228, 89.8%) and improved theoretical content (227, 89.4%). No differences were found between genders.

#### Implications for learning skills

Similarly, between 78% and 90% of respondents agreed that digital educational technology improved learning skills (Table 5), with 244 (96.1%) agreeing that it promotes self-directed learning, 233 (91.7%) agreed that it allows for the safe occurrence of errors, which in turn contributes to improving technical execution and enhanced performance in clinical skills procedures. No statistically significant differences were found based on gender or academic year.

## Discussion

The study presents the broad utilisation and constraints of digital educational technologies amongst undergraduate students, particularly within the nursing education context.

There are high levels of experience with digital educational technology and high levels of usage of common digital education technologies. The data show that the respondents reported very high use of digital educational technology, particularly the use of the institutional learning management system (Ikamva), social media for academic engagement and PowerPoint for content sharing. This aligns with previous literature highlighting how students leverage familiar, user-friendly platforms for informal learning and peer support (Wang et al. 2022), a trend that has intensified in the post-coronavirus disease (COVID) era as remote and hybrid learning environments normalised the use of digital tools for academic and social connection. The gender-based difference in social media use (χ^2^ = 14.08, *p* = 0.007) is consistent with other studies noting gendered digital behaviour, where female students often engage more in communicative and collaborative online activities (Casas, Del Rey & Ortega-Ruiz 2020). Despite the growing availability of digital educational technologies, low engagement with immersive technologies and discussion forums is notable, with only 34.3% reported using immersive technology, like VR, reflecting barriers such as access, training, or curriculum integration challenges echoed in current literature (Moreno-Ger, Burgos & Torrente 2021). Moreover, evidence from a randomised controlled trial suggests that clinical virtual simulation can significantly improve knowledge retention, clinical reasoning skills and student satisfaction (Padilha et al. 2019). Virtual reality has also been reported to accelerate skill acquisition and foster a low-stress learning environment (Chang & Lai 2021). The underutilisation of discussion forums, a key tool for collaborative learning, may reflect a broader shift amongst both students and educators towards more immediate and interactive communication formats, such as messaging apps or video conferencing platforms, which have become more prevalent in post-COVID digital learning environments (Wambua et al. 2025). The benefits are strongly affirmed by over 86% students, who indicated positive impacts on the educational experience, including enhanced accessibility, flexibility and integration of diverse resources. These findings mirror prior research that supports digital educational technology as an enabler of learner autonomy, personalised pacing and multimodal content engagement (Timotheou et al. 2023).

Despite the perception of benefits, students identified serious constraints, particularly technical issues (93.3%) and reduced peer interaction (78%), reinforcing concerns around digital infrastructure and the social dimension of learning (Bozkurt & Sharma 2020). Furthermore, Loureiro, Sousa and Antunes (2021) noted diminished interpersonal interaction and technical issues as notable drawbacks of digital educational technology implementation. Male respondents expressed significantly more concerns about depersonalisation (χ^2^ = 8.61, *p* = 0.003) and higher resistance to digital educational technology (χ^2^ = 9.69, *p* = 0.003), aligning with findings by Liaw, Hatala and Huang (2020), who observed that female students often exhibit higher digital learning self-efficacy. Related studies have similarly identified challenges such as insufficient IT training, limited internet access, ownership of digital devices, low IT competency and attitudinal barriers (Gause, Mokgaola & Rakhudu 2022; Singh & Masango 2020). Other barriers reported in the literature include inadequate Information and Communication Technology knowledge, unreliable electricity supply, limited availability of appropriate technological tools and insufficient training for educators in ICT use (Essel, Awuni & Mohammed 2020).

The role of digital educational technology was particularly promising in clinical simulation. Over 90% of students indicated its value in repeated practice until they develop competency in nursing education, where mastery of clinical procedures is essential. The statistically significant relationship (χ^2^ = 8.856, *p* = 0.031) suggests specific pedagogical strategies using digital educational technology simulations are particularly effective and may influence learning outcomes (Kefalis 2025). Overall, the results show that DETs enhance student engagement, autonomy and competence, particularly when integrated meaningfully into the learning process. Further benefits include the promotion of self-directed learning, provision of a safe environment for practice, and allowance for error-based learning, which contributes to improved clinical skill execution. However, technical, social and pedagogical barriers still limit full optimisation of these tools.

### Limitations

One limitation of this study is the non-probability sampling method, which may lead to selection bias. Students who are more familiar with digital tools may be more likely to participate in the survey, potentially skewing the results. Additionally, the study was conducted at a single institution, which may limit the generalizability of the findings to other nursing programmes. Future studies could use random sampling and include multiple institutions to enhance the generalizability of the results.

### Recommendations

Institutions should develop and implement a competency-based digital literacy framework that progressively builds students’ technological proficiency beyond basic tool usage towards meaningful educational engagement and addresses the prevalent technical barriers identified by students. They should promote social and collaborative learning tools to encourage the use of discussion forums and peer-collaborative platforms through curriculum design and educator facilitation. They should integrate immersive technologies to increase access to and training on virtual reality/Augmented reality (VR/AR) tools in clinical simulations, as these are underused despite their pedagogical benefits. They should provide targeted support based on demographic needs, address gender- and year-specific concerns through tailored workshop sessions to build digital confidence and reduce resistance. They should foster teacher presence in digital environments to strengthen the visibility and engagement of educators in online platforms to combat feelings of loss, particularly amongst senior students. They should conduct further research using qualitative studies to explore the reasons behind the constrains, gendered perceptions and year level differences in digital educational technology use would provide deeper insights for policy and practice.

## Conclusion

The study was undertaken to examine the extent and patterns of digital educational technology utilisation amongst undergraduate nursing students and to identify gaps between basic digital tool usage and meaningful pedagogically driven engagement in nursing education. The findings of this study indicate that the majority of undergraduate nursing students routinely utilise digital educational tools, particularly learning management systems, social media platforms such as Facebook, and presentation software including PowerPoint, primarily for accessing course materials, communication and assignment preparation. Whilst these technologies support basic academic functions, their use appears to be largely instrumental and task-oriented. In contrast, more interactive and pedagogically advanced digital tools, such as blogs, online discussion forums and immersive technologies including virtual or simulation-based learning environments, remain comparatively underutilised. This pattern suggests a discernible gap between foundational digital literacy and the meaningful integration of technology to promote higher-order learning, collaborative knowledge construction and experiential clinical simulation. The limited engagement with interactive and immersive platforms may constrain opportunities for reflective dialogue, peer collaboration, and the development of clinical reasoning skills within digitally mediated environments. Thus, the findings underscore the need for targeted institutional strategies to enhance digital competence amongst nursing students. Such strategies may include structured training programmes, integration of interactive technologies into curricula and the development of pedagogically aligned digital learning activities designed to foster critical thinking, collaboration and authentic clinical learning experiences in undergraduate nursing education. The study implies that undergraduate nursing education requires a systematic, curriculum integration of interactive and immersive digital technologies supported by faculty development, competency-based training, and a need for institutional investment to enhance meaningful engagement, collaborative learning and clinically relevant skill development.
